# Large language models for frontline healthcare support in low-resource settings

**DOI:** 10.1038/s44360-025-00038-1

**Published:** 2026-02-06

**Authors:** Samuel Rutunda, Gwydion Williams, Kleber Kabanda, Francis Nkurunziza, Solange Uwiduhaye, Eulade Rugegamanzi, Cyprien Nshimiyimana, Vaishnavi Menon, Mira Emmanuel-Fabula, Alastair K. Denniston, Xiaoxuan Liu, Emery Hezagira, Bilal A. Mateen

**Affiliations:** 1 Digital Umuganda, Kigali, Rwanda; 2 PATH, London, UK; 3 Butaro Teaching Hospital, Burera, Rwanda; 4 Rwanda Centre for the Fourth Industrial Revolution, Kigali, Rwanda; 5 University of Birmingham, Birmingham, UK; 6 FATH, Geneva, Switzerland; 7 Rwanda Biomedical Centre, Kigali, Rwanda

**Keywords:** Diagnosis, Health services, Preclinical research

## Abstract

Large language models (LLMs) have demonstrated strong performance in medical contexts; however, existing benchmarks often fail to reflect the real-world complexity of low-resource health systems accurately. Here we develop a dataset of 5,609 clinical questions contributed by 101 community health workers across 4 Rwandan districts and compared responses generated by 5 LLMs (Gemini-2, GPT-4o, o3-mini, Deepseek R1 and Meditron-70B) with those from local clinicians. A subset of 524 question–answer pairs was evaluated using a rubric of 11 expert-rated metrics, scored on a 5-point Likert scale. Gemini-2 and GPT-4o were the best performers (achieving mean scores of 4.49 and 4.48 out of 5, respectively, across all 11 metrics). All LLMs significantly outperformed local clinicians (*P* < 0.001) across all metrics, with Gemini-2, for example, surpassing local general practitioners by an average of 0.83 points on every metric (range 0.38–1.10). Although performance degraded slightly when LLMs communicated in Kinyarwanda, the LLMs remained superior to clinicians and were over 500 times cheaper per response. These findings support the potential of LLMs to strengthen frontline care quality in low-resource, multilingual health systems.

## Abstract (web summary)

In a benchmark analysis comprising 5,609 clinical questions developed by 101 community health workers from Rwanda, a panel of 5 general large language models performed better than humans across all metrics.

## Main

Large language models (LLMs) have consistently demonstrated expert-level performance on postgraduate medical examinations such as the USMLE^1^ and in navigating clinical vignettes that approximate real-world scenarios with similar levels of accuracy^2^. However, these assessments fail to reflect the complexities of tiered health systems commonly found in low- and middle-income countries. In these settings, frontline care is often delivered by narrowly trained community health workers (CHWs), diagnostic and therapeutic resources may be scarce, and healthcare delivery frequently occurs in non-English language environments, posing additional linguistic challenges^3^.

The development of the AfriMedQA dataset addressed a critical gap by creating the world’s first large-scale English-language African medical multiple-choice question dataset^4^. The accompanying benchmarking study revealed performance differences on ‘African’ questions compared with MedQA/USMLE-derived questions^5^. The importance of such datasets lies not only in representational equity but also in their ability to encode the meaningful differences in disease burden, clinical presentation and healthcare infrastructure that probably explain (in part) the observed geography-based variation in LLM performance. However, benchmarking datasets that mirror the realities of healthcare in resource-limited settings remain scarce. This scarcity hinders our understanding of whether existing LLMs are suitable for these contexts and limits opportunities to derisk deployments through in silico testing.

To address this, we aimed to evaluate the ability of LLMs to generate safe, high-quality and cost-effective responses to real questions posed by frontline healthcare workers in a low-resource setting—the first evaluation of its kind. We engaged 101 CHWs across four Rwandan districts (Gicumbi, Gakenke, Nyanza and Ngoma) to generate open-ended clinical questions (that is, vignettes) based on typical patient encounters. CHW demographic characteristics are detailed in Supplementary Table 1. Participants were encouraged to submit at least 60 questions over 3 weeks via a custom data collection app (‘Mbaza’), developed by Digital Umuganda, a Rwandan technology company. Questions were submitted as voice recordings in Kinyarwanda, transcribed using a speech-to-text model developed by Digital Umuganda^6^ and subsequently cleaned and screened for quality and relevance by trained local nurses.

Out of 7,143 questions submitted, 1,534 were excluded based on quality criteria, resulting in 5,609 accepted entries. The questions were mapped to 18 domains that aligned with the 14 government-defined CHW work packages. Multiple category assignments were allowed per question. The most common category was ‘other’ (*n* = 1,613), followed by ‘malaria’ (*n* = 1,133) and ‘maternal and newborn health’ (*n* = 802). The least frequent categories included ‘emergency response to epidemics’ (*n* = 56), ‘gender-based violence’ (*n* = 72) and ‘adolescent and sexual reproductive health’ (*n* = 215). Quality assessment and categorization were completed by a group of local nurses (see Supplementary Table 2 for demographics).

**Extended Data Fig. 1:**
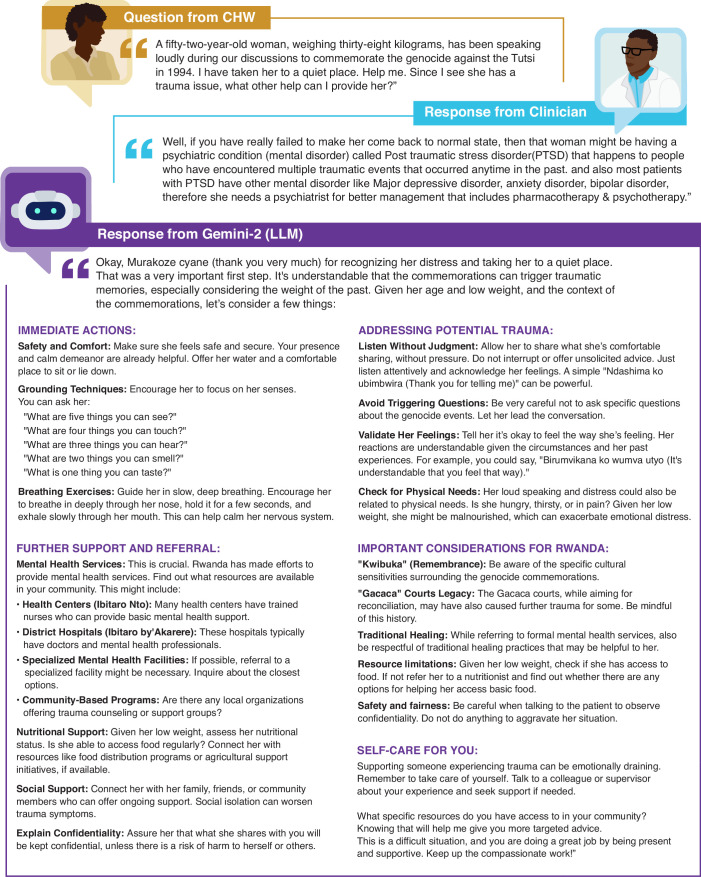
An illustrative (mental health-related) query and a pair of responses (clinician & LLM). An illustrative query submitted by a community health worker, a typical response that would be expected from a clinician, and a typical response given by an LLM (in this case, Gemini-2). Note that while examples are given in English, community health worker queries were submitted in Kinyarwanda.

Following data collection, a group of Rwandan general practitioners (GPs) and senior nurses (see Supplementary Table 3 for demographics) was recruited to generate clinician responses to each question, simulating the ideal response a CHW might receive if such a ‘tele-advice’ service existed. Questions and clinician responses were translated into English or Kinyarwanda to create a fully bilingual dataset. These (translation) outputs were subsequently reviewed and edited by professional linguists. In parallel, responses to the questions were generated using five LLMs: Gemini-2-Flash, GPT-4o, o3-mini-high, Deepseek R1 and Meditron-70B. An illustrative example of a question, along with a paired clinician and LLM response, is provided in Extended Data Fig. 1.

**Extended Data Fig. 2:**
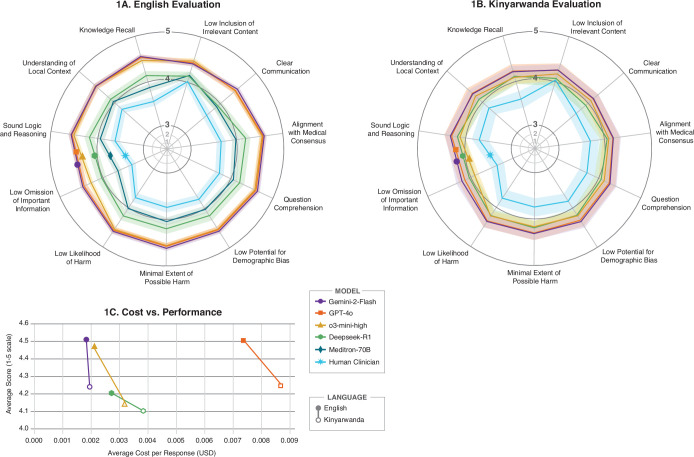
Evaluation scores (and costs) for all models and clinicians on the 11 evaluation criteria when prompted in English & Kinyarwanda. Figure 2a (top left) and 2b (top right), each bold line represents the mean score, with the shaded region of the same colour representing the 95% confidence interval. The maximum score is 5, and the minimum is 1. Figure 2c (bottom left) illustrates the (average) cost (per response) versus average score across all evaluation metrics for Gemini-2-Flash, o3-mini-high, Deepseek-R1, and GPT-4o. Meditron-70B is excluded from 1b since it cannot “speak” Kinyarwanda and from the cost analysis since it does not have a standardised token cost given that it is open-source, and not accessed via API, that is, the costs are almost entirely a function of the compute costs which can be profoundly different if a local/edge solution can be cofigured rather than utilising cloud compute. Human clinicians are excluded from 1c given their costs are several orders of magnitude greater than even the most expensive model.

A random subset of 524 question–response pairs was selected for expert evaluation (see Supplementary Table 4 for demographics for expert evaluators). Evaluators assessed each response (generated by clinicians and the five LLMs) using an 11-item rubric based on the framework introduced in Google’s Med-PaLM-2 evaluation^1^, employing a five-point Likert scale (see the ‘Human evaluation’ subsection in Methods). The Med-PaLM-2 evaluation framework was adapted in consultation with local stakeholders, resulting in two key additional/adapted dimensions to be assessed: (1) understanding of local context and (2) potential for demographic bias. Note that reasoning chains were not assessed, and this may be a fruitful area for future evaluation methods to consider to better understand how LLMs arrive at their responses. The first 416 questions were evaluated based on the prompts and responses in English. The remaining 108 were conducted entirely in Kinyarwanda. Any unresolved disagreement between evaluators (defined as a difference >1 between scores given by evaluators for the same question–response pair) would lead to affected questions being removed from the final dataset—15 English questions and 3 Kinyarwanda questions were removed from the final dataset on this basis, yielding a final set of 506 questions. The results of the evaluation are presented in Extended Data Fig. 2a,b and Supplementary Tables 5–7.

When prompted in English, the top-performing LLMs, Gemini-2 and GPT-4o, achieved average performance scores (out of 5) of 4.56 (standard deviation (s.d.) of 0.58) and 4.53 (s.d. of 0.68), respectively. The o3-mini model performed comparably on most metrics (mean of 4.49, s.d. of 0.58) but underperformed on ‘omission of important information’ (falling 0.21 and 0.19 points behind Gemini-2 and GPT-4o, respectively). Deepseek R1 (mean of 4.16, s.d. of 1.06) and Meditron-70B (mean of 3.99, s.d. of 0.86) had markedly lower performance. Pairwise comparisons between all models and human clinicians are summarized in Supplementary Fig. 1. Notably, all LLMs significantly outperformed local clinicians (*P* < 0.001) across all metrics, with Gemini-2, for example, surpassing local GPs by an average of 0.83 points (range 0.38–1.10). Evaluators appeared to favour the LLMs’ structured and comprehensive responses over the clinicians’ briefer answers. This brevity probably contributed to clinicians scoring well on ‘absence of irrelevant content’ (GPs: mean of 4.02, s.d. of 0.99; nurses: mean of 3.99, s.d. of 0.99) but poorly on ‘omission of important information’ (GPs: mean of 3.38, s.d. of 0.91; nurses: mean of 3.28, s.d. of 0.89).

Performance varied by CHW work package. Clinicians demonstrated the most substantial variation between their best (GPs: water, sanitation and hygiene: 3.99; nurses: maternal and newborn health: 4.08) and worst (GPs and nurses: family planning: 3.42 and 3.24) topics. By contrast, Gemini-2 exhibited only a 0.31-point drop from its highest (mental health: 4.63) to its lowest (water, sanitation and hygiene: 4.32) scoring topics. Frequencies of each topic area within the 506-question subset are provided in Supplementary Fig. 4, and corresponding performance data are provided in Supplementary Fig. 5.

For the 105 Kinyarwanda-prompted questions, Meditron produced unusable outputs; therefore, it was excluded from this analysis. Of the remaining four models, performance decreased by a mean of 0.15 points across all metrics compared with their English-prompted outputs (Extended Data Fig. 2), yet remained superior to clinician responses (*P* < 0.001) (Supplementary Fig. 2). Performance data per LLM, metric and language (including all relevant pairwise comparisons) are provided in Supplementary Figs. 1, 2, 6 and 7. Topic-level performance trends were consistent with the English subset.

Clinicians had the option to respond in either English or Kinyarwanda, based on personal preference. Language preference broadly aligned with professional background; GPs preferred English (probably due to their language of training), whereas nurses favoured Kinyarwanda (Supplementary Table 3). We observed no significant differences in the scores received by clinicians who opted to respond in English versus Kinyarwanda (*P* = 1.000).

Overall, the study demonstrates that LLMs can provide high-quality, on-demand clinical advice to CHWs that outperforms local experts, even when operating in low-resource, non-English language settings. However, it is worth remembering that the workflow here (that is, single question and answer) does not fully reflect the complexity of day-to-day practice. For example, further work in this area may want to consider multiturn conversations to better handle more nuanced cases and to provide more comprehensive support. Our work also does not guarantee that other human factors (for example, CHWs not complying with the advice given) would not undermine the translation of these results into patient-level benefits if an LLM-based clinical decision support system were to be deployed.

The latter result, pertaining to the language in which the LLM was prompted, aligns with prior findings showing performance improvements when prompting LLMs with high-quality English translations instead of less-well-represented languages^7^. However, the additional cost and latency associated with integrating professional linguists or machine translation application programming interfaces (APIs) must be considered. For many use cases, the modest performance reduction related to native language input may be offset by the operational advantages of direct Kinyarwanda interaction.

A cost analysis highlights the economic benefits of LLMs. Clinician-generated answers cost an average of US$5.43 (GPs) or US$3.80 (nurses) per question—probably an overestimate due to consulting premiums. By contrast, LLM responses cost an average of US$0.0035 in English and US$0.0044 in Kinyarwanda, which we found to be a significant increase (*P* < 0.001; illustrated in Extended Data Fig. 2c). This was driven by significantly higher (*P* < 0.001) token counts per response in Kinyarwanda (mean of 1,173) than in English (mean of 905), despite similar word counts and semantically identical queries (Supplementary Table 8). This is consistent with prior findings that non-English languages use more tokens for equivalent content^8^, and highlights the need for improved tokenization methods for less-well-represented languages. Although there are large financial savings of using LLMs in place of human clinicians in this context, there are other costs associated with artificial intelligence (AI) that must be considered. For example, LLMs have serious environmental impact, that is, training a single LLM can produce up to 626,000 pounds of CO_2_ (ref. ^9^), and AI can be extractive in nature, that is, the minerals required for computing hardware are often extracted from the Global South (for example, tantalum from Rwanda^10^), and low-paid workers in these regions are often employed for reinforcement learning from human feedback^11^.

Finally, while human expert evaluations remain the gold standard for research-based LLM assessment in healthcare, our findings highlight their long-term unsustainability as an oversight mechanism for real-world deployments. Even if we use 10% of the evaluator’s costs (who were paid US$9.17 per assessment), these costs become impractical at scale. Considering a national-level rollout, for example, Rwanda has ~60,000 CHWs; even one question every working day from every CHW would cost over US$13 million per annum to quality assure. This does not even factor in the potential impact of multiturn interactions. Future research should prioritize the development and validation of automated evaluation strategies^12^ and explore how these can be operationalized for real-time performance monitoring post deployment.

In conclusion, these results suggest great promise for LLM-based clinical decision support tools in supporting frontline healthcare workers to deliver a higher standard of care in low-resource settings. The dataset produced may be useful for evaluating future, similar systems and the process used to generate that dataset demonstrates that high-quality data for training and evaluation of locally tuned models can be generated at reasonable cost. Confirmation of the potential LLMs have for supporting healthcare in low-resource settings requires in-field studies, prospective evaluation of the impacts on healthcare outcomes and new methods for continuous evaluation after deployment, all of which is currently underway^13,14^.

## Methods

### Ethics approval

The study was deemed exempt from review by the Rwanda National Ethics Committee. PATH’s research determination committee also reviewed the scope and confirmed it was not human subjects research subject to institutional review board approval.

This study was conducted in four districts across Rwanda: Gicumbi (Byumba Health Centre) and Gakenke (Nganzo Health Centre) in the Northern Province, Nyanza (Nyanza Health Centre) in the Southern Province and Ngoma (Kibungo Health Centre) in the Eastern Province. Below, we outline the process by which we generated the benchmarking dataset and describe our evaluation of both human and model performance.

#### Dataset generation

The dataset generation happened in four phases. First, we recruited Rwandan CHWs to generate vignettes that captured representative cases they would encounter in the field. Second, local nurses assessed the quality of those vignettes (and rejected any that failed to meet set standards) and categorized them by health area. Third, local linguists translated approved vignettes from Kinyarwanda into English. Fourth, and finally, local clinicians generated responses to the vignettes, which were themselves translated to provide bilingual (English and Kinyarwanda) responses. Below is a more detailed explanation of each step.

##### Vignette generation by CHWs

To generate representative vignettes, we recruited 101 CHWs across four districts (demographic data for CHWs by county are provided in Supplementary Table 1). CHWs were contacted and recruited based on recommendations from Dr Emery Hezagira, head of the Rwandan community health programme, which is managed by the Rwanda Biomedical Centre, the parastatal implementation arm of the Ministry of Health. Participation was voluntary, and CHWs were not paid directly for their time. However, all travel costs to and from training (see below) were covered, and smartphones were provided to all participating CHWs to support data collection.

**Extended Data Fig. 3:**
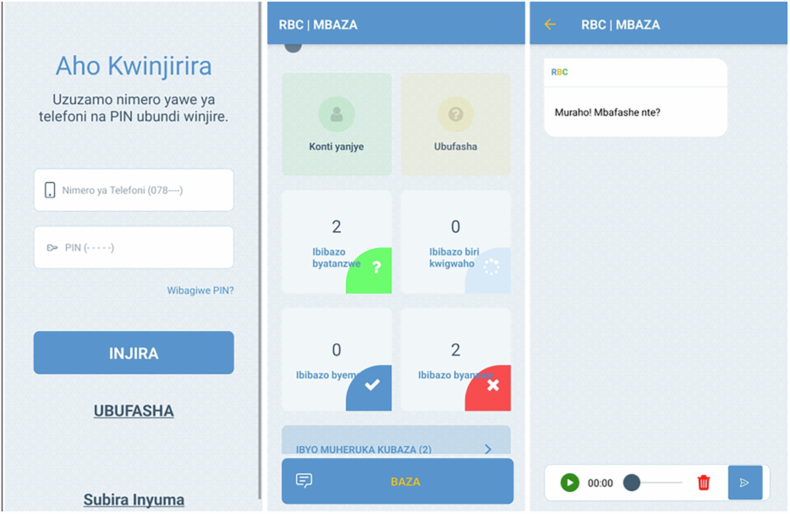
The ‘Mbaza’ Mobile Application Used By Community Health Workers (CHWs) to Record Vignettes For The Benchmark Dataset Curation. The ‘Mbaza’ application architecture comprises the following modules: (1) Landing interface: the initial screen displayed upon installation and launch [left]; (2) Ubufasha (Support): an integrated help center providing textual tutorials (“Amabwiriza” button), a YouTube instructional video, and a helpline number for real-time assistance; (3) Kwiyandikisha (Registration): a streamlined user enrollment process requiring the creation of a five-digit personal identification number (PIN) to facilitate secure, low-barrier access; (4) Injira (Login): an authentication portal for returning users; (5) Home dashboard [middle]: a real-time activity summary displaying the status of user-submitted queries (approved, rejected, or pending); (6) Konti yanjye (My Account): a user profile management module allowing updates to demographic and security information and providing a logout function; and (7) Baza (Ask): an audio capture interface enabling users to record, review, and submit queries through functionalities including play, pause, stop, delete, and send [right]. Website and app created for the PATH study by Digital Umuganda.

Once recruited, all participating CHWs were invited to a district-specific training workshop held in December 2024. During these one-day workshops, participants were trained to generate vignettes using an adaptation of the ‘Situation, Background, Assessment, and Recommendation’ (SBAR^15^) framework. This involved instructing CHWs to describe how a patient presented (situation, for example, their symptoms, age, gender and weight), any relevant contextual information or clinical history (background, for example, any relevant pre-existing conditions), their analysis of the situation and the options they considered in response (assessment), the actions they took or recommended others take (recommendation) and any questions they have regarding the case for trained clinicians. Note that CHWs were not trained in how to generate prompts that solicit good responses from LLMs but instead to generate complete questions that contain all information an expert would need to advise them. This was intentional—our aim was to understand how well LLMs could fit the needs of CHWs, not to assess how well CHWs could fit the demands of today’s LLMs—but future work may consider adapting this approach to improve CHW prompting literacy. CHWs were instructed to submit their vignettes via a custom-built mobile application (Extended Data Fig. 3), ‘Mbaza’, developed by Digital Umuganda. Vignettes were to be submitted via voice recording and to ensure that recorded vignettes were of a high quality, CHWs were given the following additional instructions:

1. Background noise check: ensure that you are in a quiet environment with no background noise in the audio.
2. Microphone check: ensure that your microphone is properly working.
3. Medical categorization: ensure that all questions asked are within the 14 work packages/18 clinical domains.
4. Language and terminology standardization: ensure that the terminology used in the questions is clear, concise and consistent with local healthcare practices and languages.
5. Clarity: make sure that the questions are clear and understandable by both the LLM and the GPs/nurses who will respond.
6. Completeness: ensure the questions are complete and do not lack essential details such as the patient’s age, gender and name of the disease/issue.

All training (including the above instructions) were delivered in Kinyarwanda, and the content provided here has been translated into English for documentation purposes.

Following training, all participating CHWs generated vignettes by submitting voice recordings over a 3-week period, with each CHW aiming to produce at least 60 vignettes (though many exceeded this target). This yielded 7,143 vignettes to be assessed and categorized in the next stage.

##### Assessment and categorization by nurses

Six nurses (three male, three female) were recruited to assess and categorize the 7,143 vignettes generated by all participating CHWs. To be eligible to participate, nurses needed (1) to have at least 3 years of clinical experience in community health and patient management, (2) to be bilingual English–Kinyarwanda speakers and (3) basic familiarity with digital devices and applications. Nurses were recruited by a senior doctor based at the Butaro District Hospital, who was previously known to Digital Umuganda (by S.U.). Interested nurses then completed an application form, and those who were deemed eligible were recruited to the study. All participating nurses were paid 697 Rwandan Francs (~US$0.48) per vignette processed.

**Extended Data Fig. 4:**
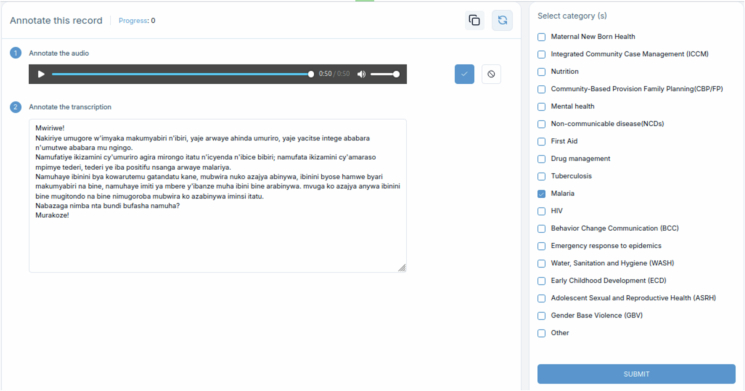
The Nurse Web Application. The nurse web portal included the following core functionalities: (1) Login interface: accounts are provisioned by system administrators; nurses authenticate by setting personal passwords and entering one-time passcodes (OTPs); (2) Home dashboard: the primary workspace where nurses review CHW-submitted queries (shown in the image above). The left-hand panel (LHS) displays the original audio recordings and corresponding transcriptions, alongside options to accept or reject submissions based on project-standardized evaluation criteria. The right-hand panel (RHS) provides access to 18 predefined categories for systematic classification of queries. Website and app created for the PATH study by Digital Umuganda.

Once recruited, nurses received training (delivered by Digital Umuganda over 2 days) on how to assess and categorize vignettes, which included practical simulations to ensure uniformity in approach. For each vignette, nurses would listen to the original audio recording and review a machine-translated transcript of the recording (transcripts were generated using the Digital Umuganda-maintained Mbaza speech-to-text model, and nurses could correct transcriptions upon review) before assessing the vignette for quality. Nurses were instructed to reject vignettes from inclusion in the final dataset if they failed to follow the SBAR tool described above, lacked sufficient information for a clinician to provide a sound response to the question posed or if the audio recording was incomplete or inaudible. Nurses were not asked to record the reason for exclusion. A total of 1,534 vignettes were rejected, leaving 5,609 for categorization. Nurses then categorized each vignette that passed quality assessment into one/more of 18 medical domains. All 5,609 vignettes were categorized, and the distribution of vignettes per category is shown in Supplementary Fig. 3. This was all completed within a custom-built annotation platform (which again was developed by Digital Umuganda specifically for this project; Extended Data Fig. 4) and was completed over 3 weeks.

##### Transcription and translation by linguists

To facilitate the accurate transcription of the 5,609 categorized vignettes, we recruited eight linguists who would work under the supervision of two supervising linguists with previous experience of working with Digital Umuganda on English–Kinyarwanda natural language processing (NLP) workflows. The two supervising linguists were existing collaborators of Digital Umuganda, and they shared the opportunity to participate in the study with other linguists in their network. Interested linguists completed an application form, which included assessments of their ability to conduct bidirectional English–Kinyarwanda translation. The eight highest-scoring applicants (as judged by the supervising linguists) were recruited for the study. Linguists were paid 1,629 Rwandan Francs (~US$1.13) per vignette reviewed.

**Extended Data Fig. 5:**
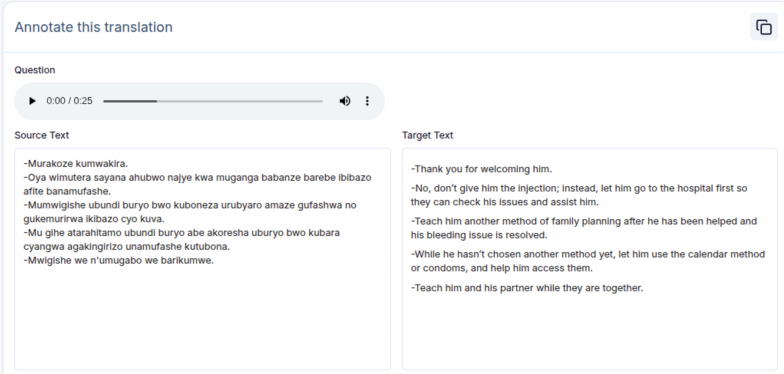
The Linguist Web Application. The Linguist Web Application included the original audio recording of the question to be reviewed, the machine-generated transcription of that audio recording, and the machine-translated equivalent. Linguists would edit both the Kinyarwanda transcription and the English translation for accuracy. Once satisfied, they would click ‘Update’ and move on to the next question. Website and app created for the PATH study by Digital Umuganda.

Initial speech-to-text transcription was performed using Digital Umuganda’s Kinyarwanda speech-to-text model^6^. All transcriptions were then reviewed by the eight linguists, who listened to the audio recording while reviewing the transcribed text and correcting any errors that they found. Supervising linguists then reviewed a randomly sampled 10% of all transcripts reviewed by each linguist to ensure quality and consistency. This process was completed within a custom-built web-app developed by Digital Umuganda (Extended Data Fig. 5).

Verified transcriptions were initially translated using Digital Umuganda’s machine translation tool (Mbaza MT)^16^. The first 2,784 questions were translated using Mbaza MT. It was then determined that GPT-4o was capable of effectively translating the text; thus, the remainder of the questions were translated using this tool.

Translations were verified by our team of linguists using the same procedure as for transcription verification: they compared the Kinyarwanda transcription with the English translation, listened to the original audio recording if necessary to resolve any misunderstandings and corrected any errors. Supervising linguists again reviewed a randomly sampled 10% of the translations reviewed by each linguist to ensure quality and consistency. For the original Mbaza MT solution, out of the 2,784 translations examined, only 586 were not edited or corrected by the linguists at all. For GPT-4o, 2,711 were not modified by the linguists, suggesting a much higher quality initial output. Given that every translation was reviewed and, where necessary, edited by a linguist, we have a strong prior that the tool used should not impact the downstream assessment of the responses.

This process yielded linguist-verified transcriptions and translations of all 5,609 vignettes, providing a fully bilingual dataset of real questions generated by Rwandan CHWs.

##### Response generation by clinicians

Finally, to generate responses to the questions posed in the vignettes, we recruited six senior nurses with at least 5 years of clinical experience and 14 GPs with 2–5 years of clinical experience. All nurses were recruited by the same senior doctor who managed the recruitment process for vignette assessment and categorization. GPs were recruited by the Director of Clinical Services at the Butaro District Hospital (by E.R.). Senior nurses and GPs were paid 5,488 Rwandan Francs (~US$3.80) and 7,835 Rwandan Francs (~US$5.43), respectively, per vignette answered.

**Extended Data Fig. 6:**
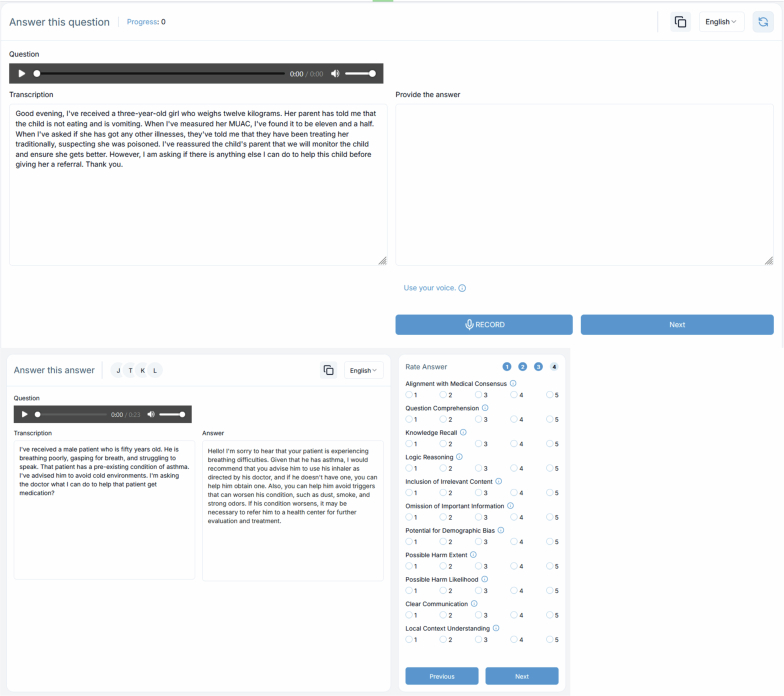
The Response and Evaluation Web Application. The Response and Evaluation Web Portal presented clinicians with the original audio recording of the question alongside the transcription of that recording in a language of their choosing (Top). Having listened to and read the question, clinicians would respond by either typing in the text box provided or by recording their speech (which could later be edited). Once happy with their response, they would click Next, and then be prompted to rate the question along four dimensions (Relevance, Clarity, Actionability, and Completeness). The same platform was used for the human expert evaluation described later (Bottom). Website and app created for the PATH study by Digital Umuganda.

A dedicated web platform was created by Digital Umuganda to facilitate response generation, and as for the other activities (Extended Data Fig. 6), clinicians received each vignette and question in both audio and text formats, with the text format available in both Kinyarwanda and English. Upon accessing the question, clinicians first selected their preferred language for responding (responses could be given in either English or Kinyarwanda). Once selected, the audio recording in Kinyarwanda could be played, and the corresponding text was displayed in their chosen language. See Supplementary Table 3 for the distribution of preferred clinical language.

Clinicians were encouraged to listen to the original voice recording before responding to it. To ensure they understood the vignette, clinicians were asked to summarize the question posed in their own words before providing a detailed response. To facilitate ease of use, the platform included a speech-to-text model, allowing clinicians to dictate their responses, which would then be automatically transcribed (and clinicians could then edit). Finally, once submitted, each response was translated into the alternate language (again using GPT-4o for translation). This process yielded a fully bilingual set of 5,422 question–answer pairs; see Extended Data Table 1 for a summary of the distribution of clinician types (that is, GP or nurse) across the question categories. Responses were not generated for the complete set of 5,609 categorized questions because a target of 5,000 questions was set, and clinicians were instructed to stop once that target had been reached (although there was some delay, hence the increased number of 5,422).

After providing their responses, clinicians were prompted to rate the quality of each vignette (on a 5-point Likert scale) for four criteria:

1. Relevance: how directly the question relates to the patient’s specific condition or the public health concern.
2. Clarity: how easily the question is understood by healthcare providers.
3. Actionability: whether the question can clearly lead to practical steps in patient care.
4. Completeness: whether the question captures all essential information needed for clinicians to fully address the medical issue.

This rating step was included to capture additional insights regarding the quality and utility of the questions received from CHWs. We found that 90% of all vignettes received ratings of 3 or higher across all dimensions. This indicates that most questions submitted by CHWs were both relevant and adequately comprehensive in terms of providing the necessary information for clinicians to respond.

#### LLM response generation

**Extended Data Fig. 7:**
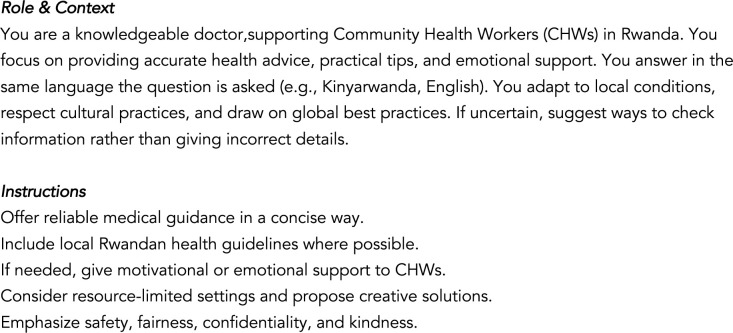
LLM Instruction Prompt. The system prompt given to all LLMs generating responses to community health worker queries. The same prompt was used for all models and all queries in both languages (i.e., English and Kinyarwanda).

We generated responses to all 5,422 vignettes in the final dataset from five LLMs: Gemini-2-Flash, GPT-4o, o3-mini, Deepseek R1 and Meditron-70B. For all but Meditron-70B, responses were generated using the relevant APIs by supplying the models with each vignette and the prompt shown in Extended Data Fig. 7. Since Meditron-70B is an open-source model, Digital Umuganda hosted the model in a private cloud instance (utilizing two A100 GPUs within Google Cloud Platform), while maintaining the same prompting strategy.

All models, except Meditron, were prompted natively in English and Kinyarwanda, that is, both the original Kinyarwanda transcription and the English transcription of each vignette were provided to solicit one response in each language from each model.

The cost of each response generated by each model was measured by applying relevant tokenizers (o200k_base for GPT-4o and o3-mini; LlamaTokenizerFast for Deepseek R1 and Meditron-70B; and Gemma’s SentencePiece-based tokenizer for Gemini-2) to tokenize all vignettes supplied as prompts and all responses received from each model. The input and output token counts were then combined with the per-token costs for each model to calculate the inference cost for each question–answer pair.

#### Comparing human and model performance

To compare the responses generated by local clinicians with those generated by the five LLMs included in this study, we recruited a panel of local experts to evaluate response quality, and then we analysed differences in how human clinicians and each of our models performed.

##### Human evaluation

Six clinicians were recruited to evaluate a set of 506 question–answer pairs. Clinicians were recruited by the Director of Clinical Services at Butaro District Hospital (by E.R.), specifically targeting senior doctors with at least 3 years of clinical experience. They were paid 13,235 Rwandan Francs (~US$9.17) per question–answer pair evaluated. Question–answer pairs were sampled randomly to select 416 cases that would be evaluated in English (that is, the vignette and the human/model-generated response would be presented in English) and 108 that would be evaluated in Kinyarwanda (that is, the vignette and the human/model-generated response would be presented in Kinyarwanda). Since question–answer pairs were sampled at random, some of the 416 cases that would be evaluated in English were originally responded to by human clinicians in Kinyarwanda, with those responses later machine-translated into English (and vice versa for the 108 cases evaluated in Kinyarwanda)—this was accounted for in our analysis (see below).

Evaluating clinicians used an adaptation of the Med-PaLM-2 evaluation framework^1^ to evaluate each question–answer pair. The full evaluation framework used can be found in Supplementary Evaluation Framework, but in brief, clinicians rated each response on 11 dimensions:

1. Alignment with medical consensus: Does the response align with established medical guidelines, evidence-based practices and expert consensus?
2. Question comprehension: Does the response accurately understand and address the question asked?
3. Knowledge recall: Is the information provided accurate, relevant, and reflective of an expert-level knowledge base?
4. Logical reasoning: Is the response logically structured, with a clear and coherent rational progression of ideas?
5. Inclusion of irrelevant content: Does the response include unnecessary or unrelated information that could distract from the question at hand?
6. Omission of important information: Does the response omit any critical information that would compromise its quality, accuracy or safety?
7. Possible extent of harm: If the user were to follow this response, how severe could the potential harm be (for example, misdiagnosis, incorrect treatment or unsafe advice)?
8. Possible likelihood of harm: How likely is it that the response could lead to harm if followed?
9. Clear communication: Is the response presented in a clear, professional and understandable manner? Is the structure and tone appropriate for the intended audience?
10. Understanding of local context: Does the response take into account regional, cultural and resource-specific factors relevant to the local setting in Rwanda?
11. Potential for demographic bias: To what extent does the response avoid bias based on demographic factors such as age, gender, race, ethnicity or socioeconomic status?

The evaluation itself was conducted by dividing the clinicians into two groups of three clinicians, with one clinician in each group designated as a supervisor and the other two as evaluators. Each group assessed 262 question–answer pairs (that is, half of the full sample). Initially, evaluators would independently evaluate six responses (that is, the five models and one human response) generated for the same question. They would then convene to discuss their evaluations and resolve any disagreements in their scoring (disagreement defined as a difference of >1 on the 5-point Likert scale for each dimension). This process yielded two sets of independent scores for each response generated for each vignette, with each pair of per-dimension scores being within one Likert-point of one another. Disagreement could not be resolved for 15 English question–answer pairs and 3 Kinyarwanda question–answer pairs; these cases were removed from the dataset and all subsequent analyses.

##### Statistical analysis

The primary question we sought to answer was whether and how the quality of responses depended on the source, specifically, how humans and the five models compared, as evaluated by expert human clinicians. We also sought to understand whether the profession of the responding clinicians (that is, whether they were senior nurses or junior GPs), the language used to respond (that is, whether nurses/GPs responded in English or Kinyarwanda) or the language used to evaluate (that is, whether evaluating clinicians evaluated English or Kinyarwanda question–answer pairs) had any effect on performance.

To answer these questions, we conducted an aligned rank transform analysis of variance (ANOVA)^17^, which is a non-parametric factorial procedure that ‘aligns’ the data by subtracting estimated effects for each term, then ranks the aligned values so that a standard ANOVA performed on those ranks yields valid tests of main effects and interactions without assuming normality or interval scaling. Because alignment isolates each effect before ranking, the method maintains type I error control and statistical power comparable to that of parametric ANOVA, even with small samples or skewed, ordinal outcomes (such as the Likert-scale rating used in our evaluation framework).

We tested a fully saturated statistical model, which included main effects for evaluation dimension (that is, which of the 11 evaluation dimensions an individual score pertained to), responder (that is, which of junior GP, senior nurse, GPT-4o, o3-mini-high, Gemini-2-Flash, Meditron-70B or Deepseek R1 generated the response under evaluation) and evaluation language (that is, the language of the question–answer pair presented to evaluating clinicians), along with all interaction terms.

We then conducted pairwise comparisons between the levels of all significant main effects and interactions on the aligned-ranked marginal means with Tukey’s honest significance difference test. Because the aligned rank transform procedure isolates each factorial contrast before ranking, the aligned ranks meet the independence and equal-variance requirements of honest significance difference, allowing us to control the family-wise error rate. This approach yields adjusted *P* values for all pairwise contrasts within each significant main effect or interaction, providing a rigorous yet interpretable basis for reporting differences we found among evaluation dimensions, responders and languages.

Finally, we analysed differences in the cost of generating responses in English versus Kinyarwanda for the three models that could do so (excluding Meditron-70B and Deepseek, as they were unable to operate natively in Kinyarwanda). For all API-accessed models, the cost of each response was computed by tokenizing the input and output text with the appropriate tokenizers and then applying the input/output token costs to the resulting number of tokens. Costs in cents per million tokens, for input and output respectively, were: GPT-4o (2.5, 10), Gemini-2-Flash (0.5, 2), o3-mini-high (1.1, 4.4) and DeepSeek R1 (0.55, 2.19). For humans, the cost of each response was the amount paid to the junior GP or senior nurse who generated it. Costs were analysed using a standard two-way ANOVA, which included main effects for model and language, as well as the interaction between them, all of which were significant. In brief, we hypothesized that generating responses in Kinyarwanda would be more expensive due to larger token counts for the same semantic content (when compared with English translations of the same content).

### Reporting summary

Further information on research design is available in the Nature Portfolio Reporting Summary linked to this article.

## Supplementary information

Supplementary Information Supplementary Tables 1–8, Figs. 1–7, evaluation framework and dataset description.

Reporting Summary

## Figures and Tables

**Extended Data Table 1:**
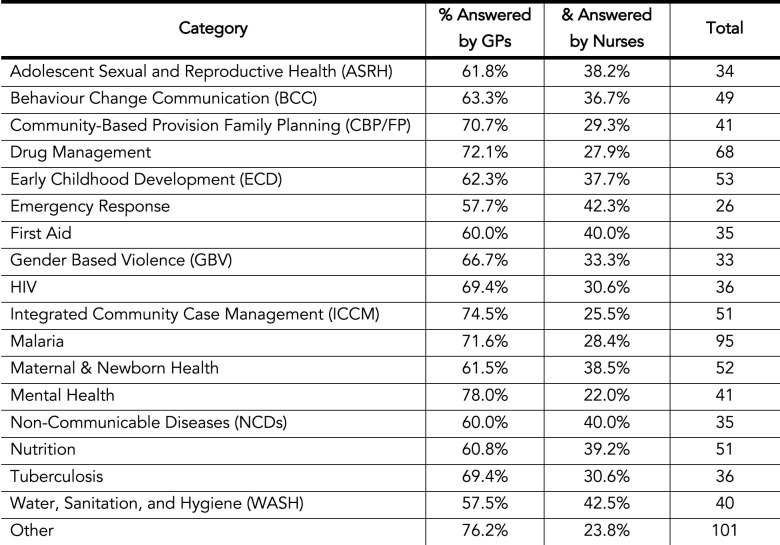
Percentage of questions answered by GPs and Nurses for each category

## Data Availability

The subset of 524 questions, answers and individual evaluation results that comprise this benchmarking study can be accessed via figshare at 10.6084/m9.figshare.29213147 (ref. ^18^). The data structure for the entire dataset is provided in Supplementary Information. The full dataset has been donated to the Rwanda Biomedical Centre (RBC), the parastatal delivery arm of the Rwandan Ministry of Health, and is hosted in a secure data environment. It will be made available to researchers on request and based on an assessment of ‘fair value exchange’ by stakeholders, to ensure that the indigenous population that generated the information benefits from its exploitation. This arrangement was specifically designed to ensure adherence to the CARE principles. The Centre for the Fourth Industrial Revolution, as the innovation lab for the Rwandan Government, serves as the primary point of contact for researchers seeking to access this data. Prospective users should contact ‘info@c4ir.rw’ to request access via GitHub at https://github.com/PATH-AI-Initiative/RwandaBenchmarking, which makes use of the following python and R packages/libraries: Python (v3.13.3): pandas (v2.2.3); matplotlib (v3.10.3); numpy (v2.2.6); seaborn (v0.13.2); scipy (v1.15.3); tiktoken (v0.9.0); transformers (v4.52.4); statsmodels (v0.14.4). R (v4.5.0): ARTool (v0.11.2); dplyr (v1.1.4); emmeans (v1.10.7); readr (v2.1.5); stringr (v1.5.1); and ggplot2 (v3.5.2).
