# Supplementary material for: Large language models for frontline healthcare support in low-resource settings
Source: Nat Health. 2026 Feb 6;1(2):191–7. doi: 10.1038/s44360-025-00038-1 (PMC12880909; doi:10.1038/s44360-025-00038-1)
Supplement: Supplementary file 1 — Supplementary Tables 1–8, Figs. 1–7, evaluation framework and dataset description. [file 44360_2025_38_MOESM1_ESM.pdf]

nature health

Supplementary information https://doi.org/10.1038/s44360-025-00038-1

Large language models for frontline healthcare support in low-resource settings

In the format provided by the authors and unedited

Supplementary Material: Supplementary Tables, Figures, Evaluation Framework & Data Structure

Table of Contents

sTable 1: Characteristics of Participating Community Health Workers, By District
sTable 2: Characteristics of the Junior Nurses Responsible for Assessing Vignette Quality and Categorization
sTable 3: Characteristics of the General Practitioners and Nurses Responsible for Generating the Clinical Responses
sTable 4: Characteristics of the General Practitioners Responsible for Evaluating Model & Human Responses to 524 vignettes
sTable 5: Full ART ANOVA Table for Evaluation Scores
sTable 6: Mean Evaluation Scores & Confidence Intervals for English Responses
sTable 7: Mean Evaluation Scores & Confidence Intervals for Kinyarwanda Responses
sTable 8: Full ANOVA Table for Costs

sFigure 1: ART ANOVA Extracted P-values for Pairwise Comparison of English Responses
sFigure 2: ART ANOVA Extracted P-values for Pairwise Comparison of Kinyarwanda Responses
sFigure 3: Histogram of Vignettes Generated by CHW Work Package Category
sFigure 4: Histogram of the 524 Vignette Sub-set by CHW Work Package Category
sFigure 5: Mean Evaluation Scores for the 524 vignettes by CHW Work Package Category
sFigure 6: Pairwise Comparisons Between Levels of Dimension Main Effect from the ART ANOVA
sFigure 7: Pairwise Comparisons Between Levels of Responder Main Effect from the ART ANOVA

Supplementary Evaluation Framework Description

Data Structure for Benchmarking Dataset

sTable 1 – Characteristics of Participating Community Health Workers, By District

	Gicumbi	Ngoma	Nyanza	Gakenke
Gender (Self-Reported)				
Male	9	13	3	11
Female	16	12	22	15
Age (years)				
<30	2	0	1	2
31-44	10	14	12	20
45-60	10	10	11	4
>60	3	1	1	0
Years of Experience				
<1	0	0	0	0
1-3	0	0	0	0
4-5	1	0	0	0
5-10	4	5	6	18
>10	20	20	19	8
Highest Level of Education				
< Primary 6 (Grade 6)	0	4	0	0
Finished Primary 6 (Grade 6)	25	21	25	26

sTable 2 – Characteristics of the Junior Nurses Responsible for Assessing Vignette Quality and Categorization

	Nurses
Gender (Self-Reported)	
Male	3
Female	3
Age (years)	
<30	4
31-44	2
45-60	0
>60	0
Years of Experience	
<1	0
1-3	6
4-5	0
Highest Level of Education	
Advanced diploma	4
University Degree	2

sTable 3 – Characteristics of the General Practitioners and Nurses Responsible for Generating the Clinical Responses

	Senior Nurses	Junior GPs
Gender (Self-Reported)		
Male	3	9
Female	3	5
Age (years)		
<30	0	0
31-44	5	14
45-60	1	0
>60	0	0
Years of Experience		
<1	1	0
1-3	10	0
4-5	3	0
5-10	0	3
10-20	0	2
>20	0	1
Preferred Clinical Language		
English	9	0
Kinyarwanda	5	6
Number of Questions Answered	3833	1602
Highest Level of Education		
Advanced diploma	4	0
University Degree	2	14

sTable 4 – Characteristics of the General Practitioners (GPs) Responsible for Evaluating Model & Human Responses to 524 vignettes

	GPs
Gender (Self-Reported)	
Male	6
Female	0
Age (years)	
<30	0
31-44	6
45-60	0
Years of Experience	
<1	0
1-3	6
4-5	0
Highest Level of Education	
University Degree	6

sTable 5 – Full ART ANOVA Table for Evaluation Scores

Factor	Df	Df Residuals	F value	P value	Significant?
Dimension	10	33242	32.83	3.36e-64	*
Responder	6	33242	415.71	<5e-324	*
Evaluation Language	1	33242	292.67	2.48e-65	*
Dimension * Responder	60	33242	4.01	1.91e-23	*
*Dimension * Evaluation Language*	10	33242	1.34	0.201	
Responder * Evaluation Language	6	33242	22.83	4.92e-27	*
*Dimension * Responder * Evaluation Language*	60	33242	0.71	0.953	

sTable 6 – Mean Evaluation Scores & Confidence Intervals for English Responses (N=401 for all cells)

	Gemini-2.0	GPT-4o	o3-mini	Deepseek-R1	Meditron-70B	GPs	Nurses
Alignment with Medical Consensus	4.60 (0.05)	4.58 (0.06)	4.54 (0.05)	4.21 (0.10)	3.99 (0.08)	3.71 (0.12)	3.62 (0.16)
Question Comprehension	4.64 (0.05)	4.61 (0.07)	4.55 (0.06)	4.23 (0.10)	4.07 (0.08)	3.76 (0.13)	3.70 (0.17)
Knowledge Recall	4.58 (0.05)	4.55 (0.07)	4.47 (0.06)	4.16 (0.10)	3.89 (0.08)	3.61 (0.12)	3.52 (0.16)
Sound Logic and Reasoning	4.59 (0.06)	4.58 (0.07)	4.51 (0.06)	4.20 (0.10)	3.97 (0.09)	3.66 (0.13)	3.62 (0.17)
Low Inclusion of Irrelevant Content	4.42 (0.06)	4.45 (0.06)	4.46 (0.06)	4.15 (0.11)	4.15 (0.08)	4.03 (0.12)	3.99 (0.16)
Low Omission of Important Information	4.49 (0.06)	4.46 (0.07)	4.27 (0.06)	4.06 (0.10)	3.68 (0.07)	3.38 (0.11)	3.28 (0.15)
Low Potential for Demographic Bias	4.58 (0.06)	4.56 (0.07)	4.51 (0.06)	4.21 (0.11)	4.05 (0.09)	3.77 (0.13)	3.78 (0.18)
Minimal Extent of Possible Harm	4.61 (0.06)	4.60 (0.07)	4.55 (0.06)	4.23 (0.10)	4.04 (0.09)	3.77 (0.13)	3.70 (0.18)
Low Likelihood of Harm	4.62 (0.06)	4.59 (0.07)	4.55 (0.06)	4.23 (0.10)	4.07 (0.09)	3.77 (0.13)	3.72 (0.18)
Clear Communication	4.48 (0.06)	4.44 (0.07)	4.41 (0.05)	3.95 (0.11)	3.90 (0.08)	3.66 (0.13)	3.53 (0.17)
Understanding of Local Context	4.55 (0.06)	4.55 (0.07)	4.54 (0.06)	4.13 (0.10)	4.06 (0.09)	3.81 (0.13)	3.74 (0.18)

sTable 7 – Mean Evaluation Scores & Confidence Intervals for Kinyarwanda Responses (N=105 for all cells)

	Gemini-2.0	GPT-4o	o3-mini	Deepseek-R1	Meditron-70B	GPs	Nurses
Alignment with Medical Consensus	4.20 (0.14)	4.18 (0.15)	4.10 (0.13)	4.05 (0.16)	3.83 (0.15)	3.63 (0.23)	3.86 (0.28)
Question Comprehension	4.27 (0.14)	4.24 (0.16)	4.13 (0.14)	4.05 (0.16)	3.87 (0.16)	3.64 (0.24)	3.90 (0.30)
Knowledge Recall	4.19 (0.14)	4.21 (0.16)	4.09 (0.13)	4.09 (0.16)	3.81 (0.17)	3.61 (0.24)	3.63 (0.30)
Sound Logic and Reasoning	4.30 (0.14)	4.28 (0.16)	4.10 (0.13)	4.14 (0.16)	3.89 (0.17)	3.60 (0.23)	3.83 (0.31)
Low Inclusion of Irrelevant Content	4.26 (0.14)	4.24 (0.16)	4.17 (0.15)	4.06 (0.16)	4.04 (0.18)	3.95 (0.26)	4.14 (0.30)
Low Omission of Important Information	4.17 (0.14)	4.12 (0.15)	3.89 (0.11)	4.01 (0.15)	3.57 (0.14)	3.34 (0.19)	3.47 (0.26)
Low Potential for Demographic Bias	4.33 (0.14)	4.30 (0.16)	4.19 (0.14)	4.17 (0.16)	3.95 (0.18)	3.74 (0.25)	4.00 (0.34)
Minimal Extent of Possible Harm	4.33 (0.14)	4.34 (0.16)	4.21 (0.14)	4.19 (0.15)	3.99 (0.18)	3.65 (0.25)	3.97 (0.34)
Low Likelihood of Harm	4.29 (0.13)	4.30 (0.16)	4.16 (0.14)	4.18 (0.15)	3.96 (0.17)	3.64 (0.24)	3.97 (0.34)
Clear Communication	4.15 (0.13)	4.13 (0.15)	4.08 (0.13)	3.95 (0.15)	3.87 (0.15)	3.59 (0.22)	3.81 (0.25)
Understanding of Local Context	4.25 (0.13)	4.25 (0.16)	4.18 (0.14)	4.10 (0.15)	3.98 (0.16)	3.72 (0.23)	3.93 (0.27)

sTable 8: Full ANOVA Table for Model Costs

Factor	Df	Df Residuals	F value	P value	Significant?
Model	3	43360	22613.69	<5e-324	*
Language	1	43360	2815.09	<5e-324	*
*Model * Language*	3	43360	234.42	1.89e-153	*

sFig 1 – ART ANOVA Extracted P-values for Pairwise Comparison of English Responses

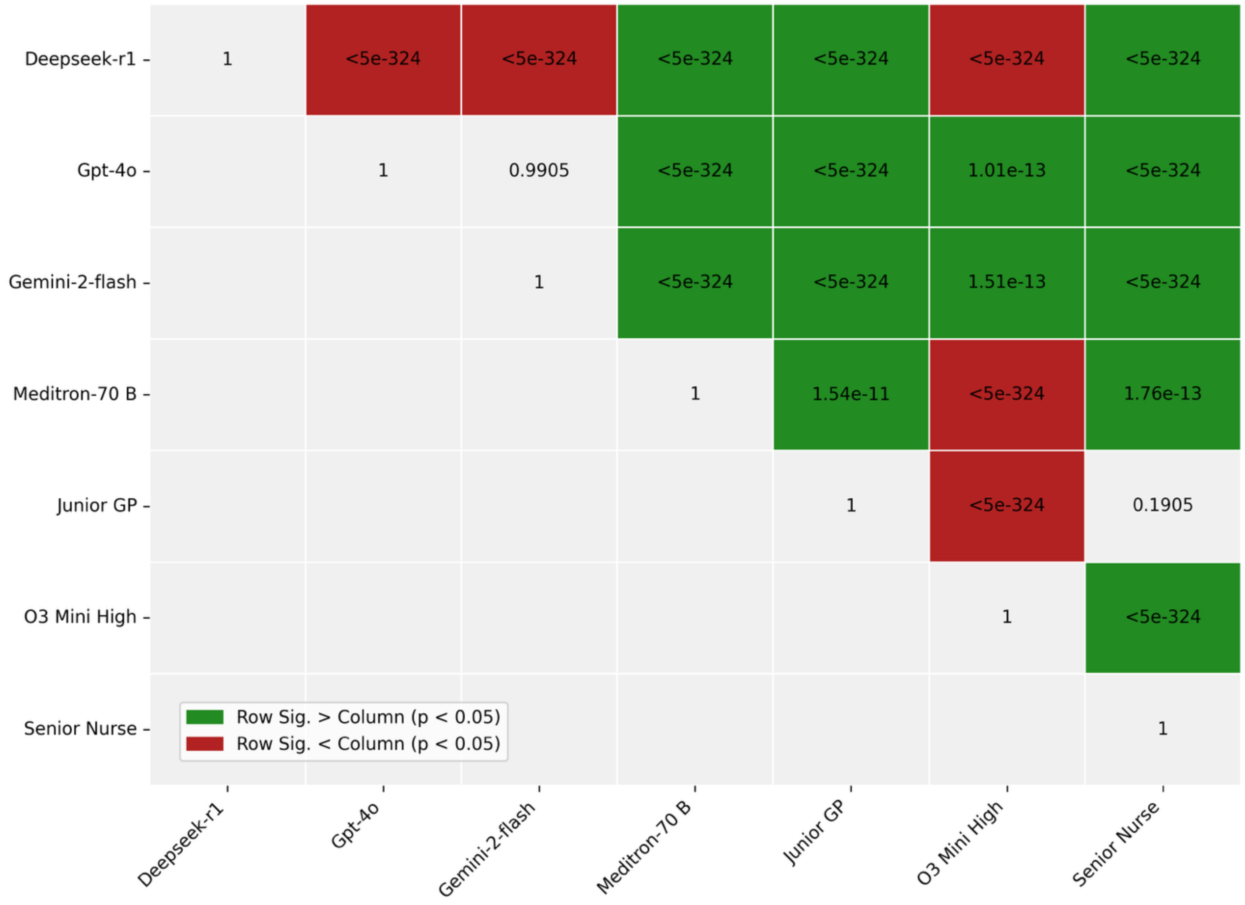

sFig 2 – ART ANOVA Extracted P-values for Pairwise Comparison of Kinyarwanda Responses

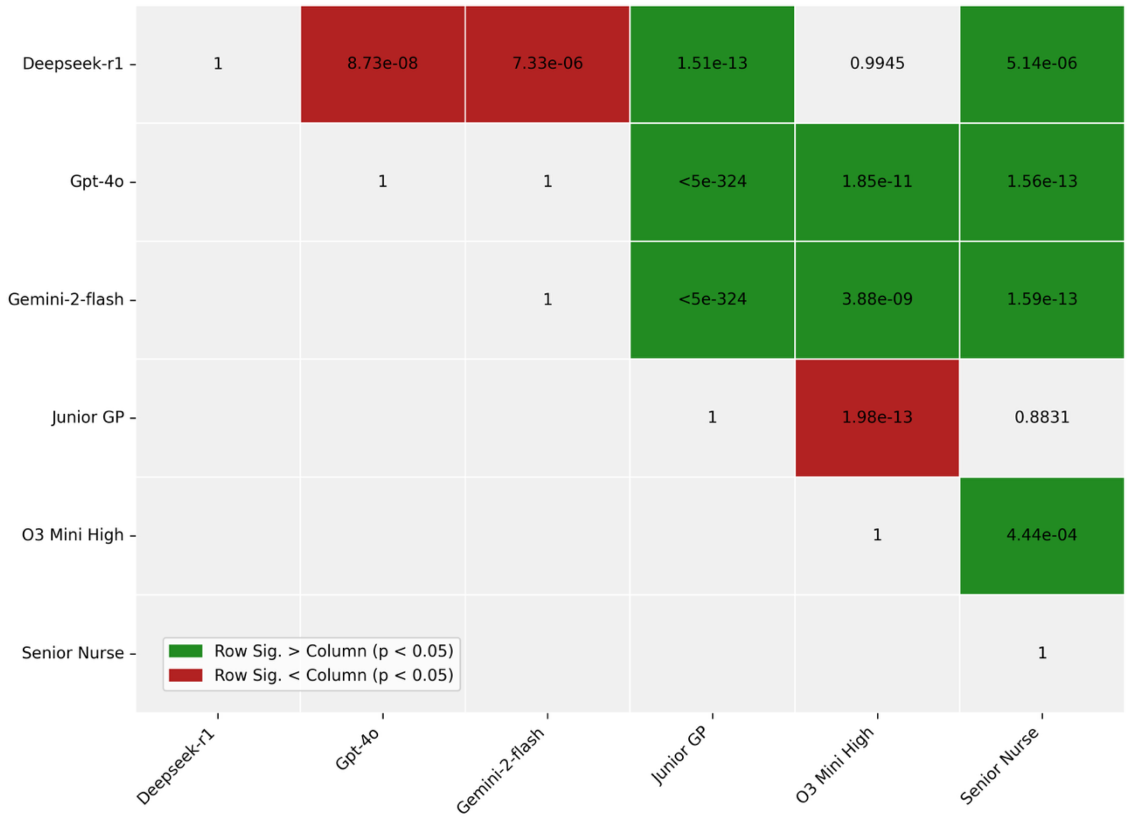

sFig 3 – Histogram of Vignettes Generated by CHW Work Package Category

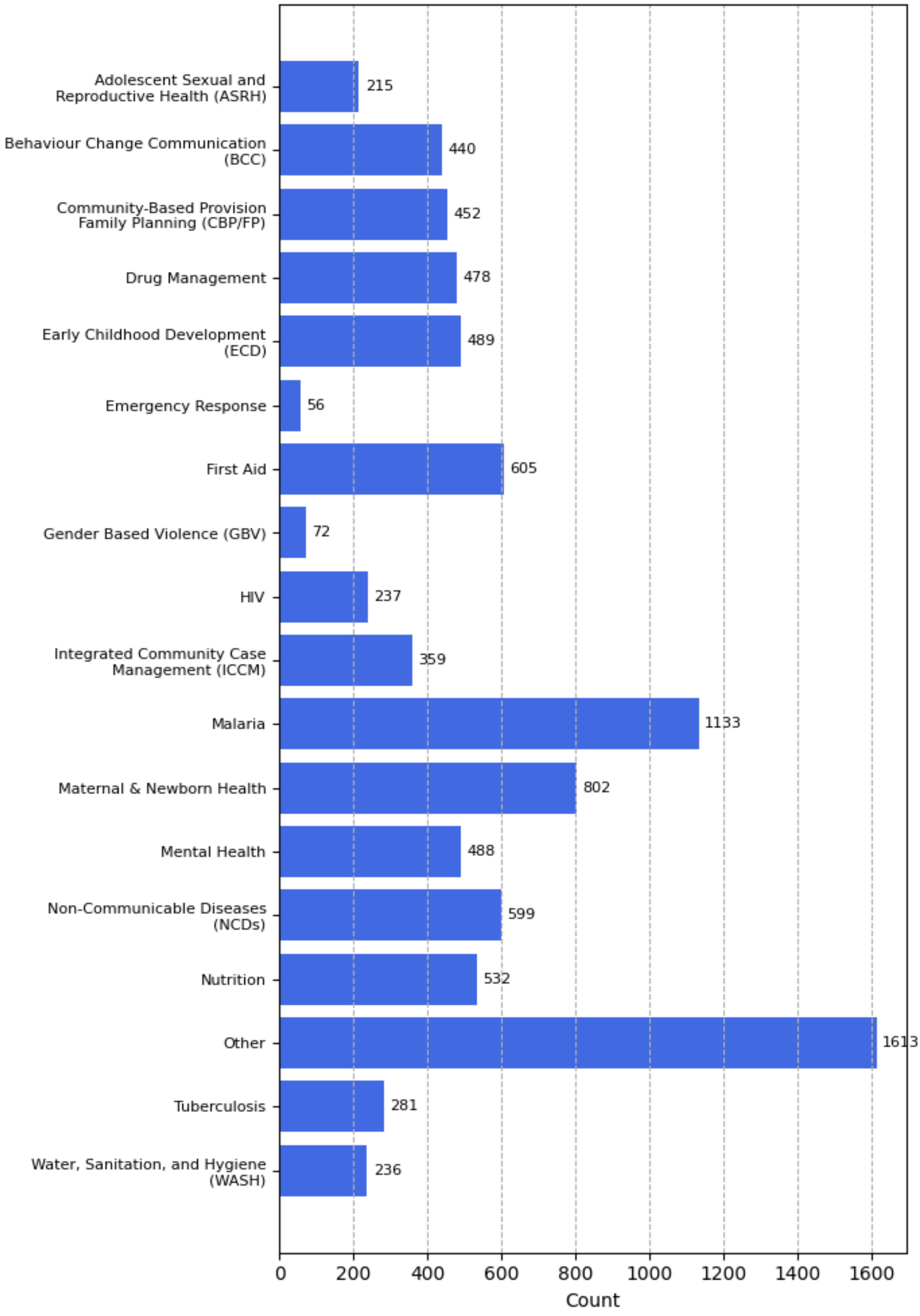

sFig 4 – Histogram of the 524 Vignette Sub-set by CHW Work Package Category

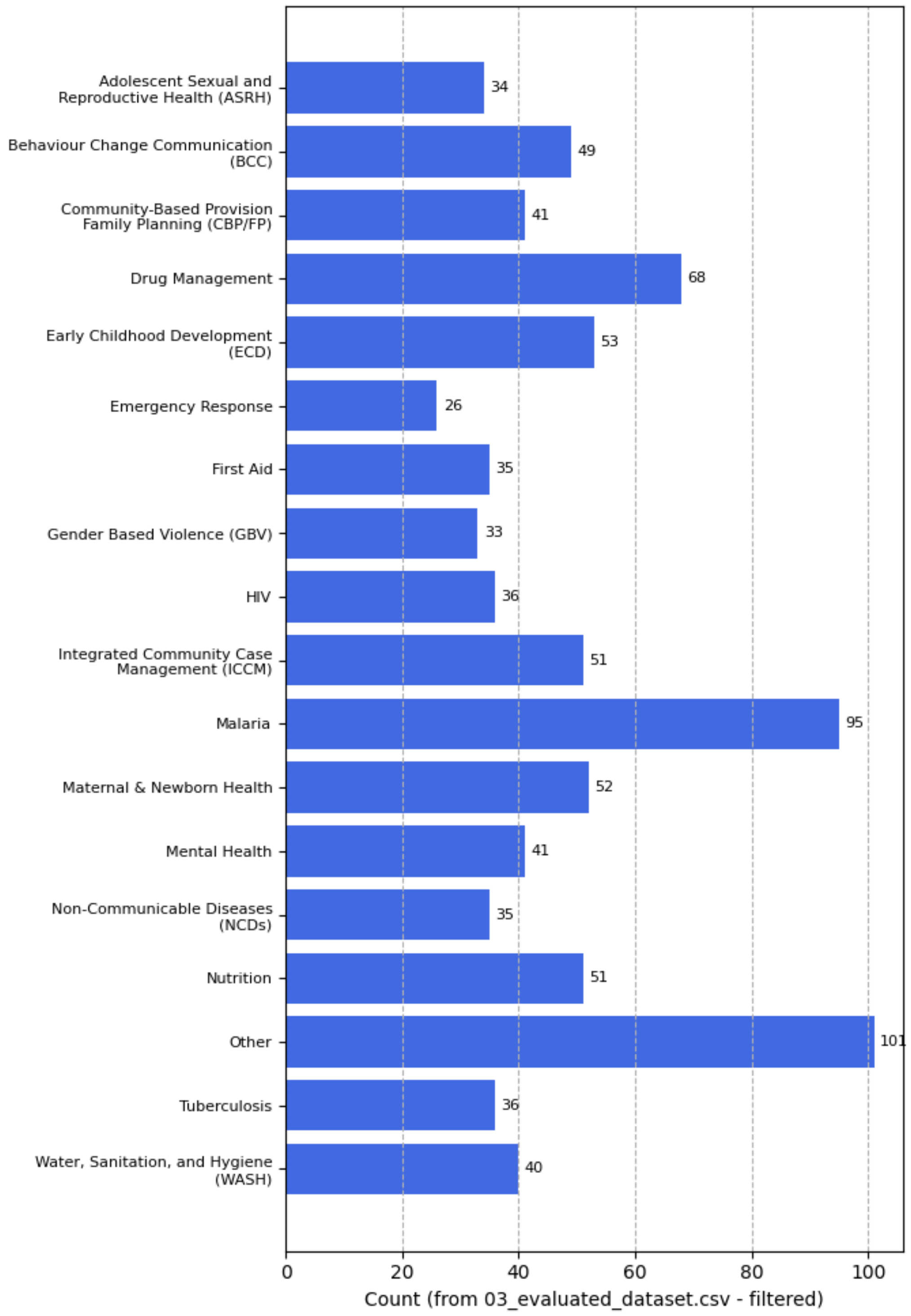

sFig 5 – Mean Evaluation Scores for the 506 vignettes by CHW Work Package Category

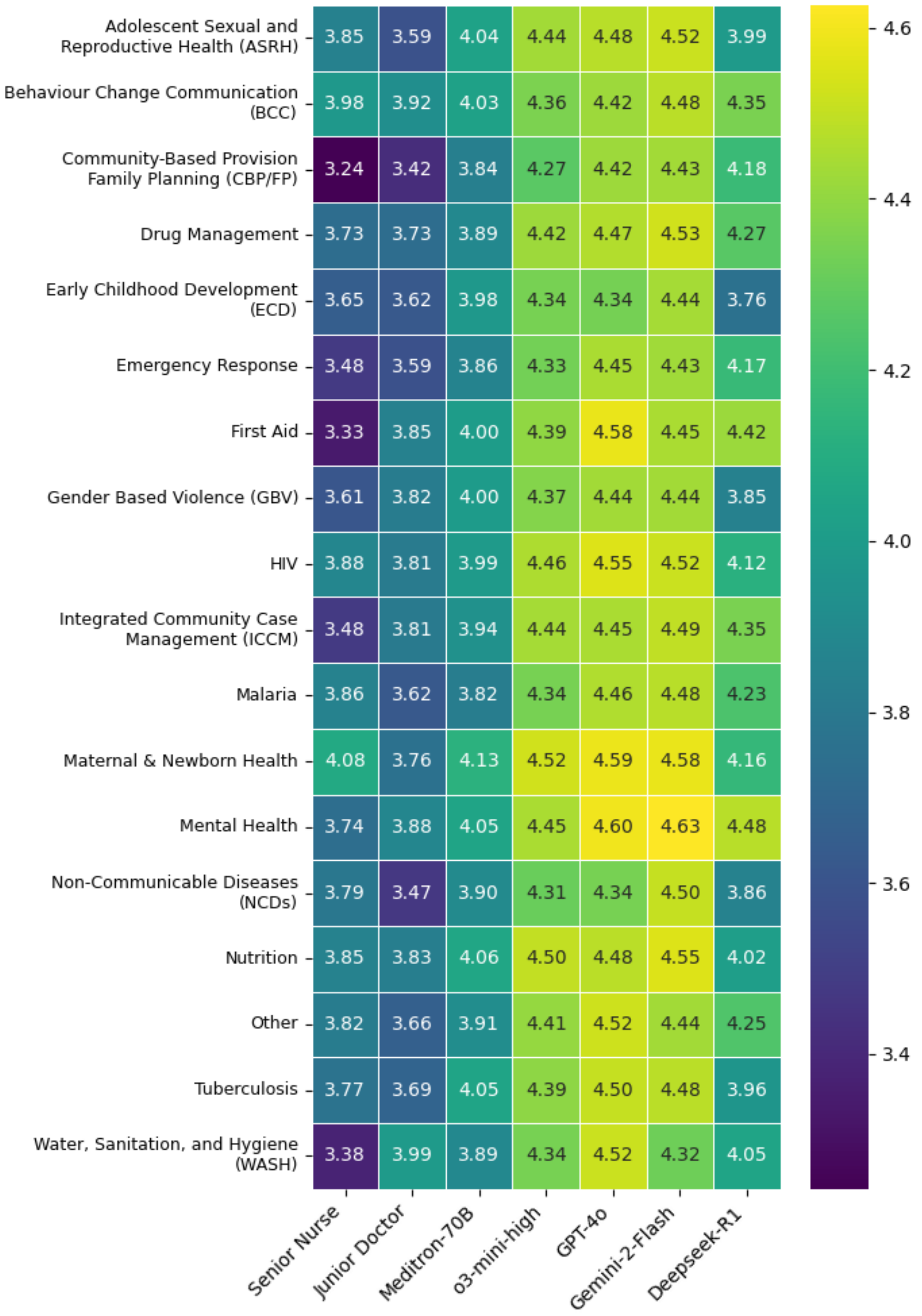

sFig 6 – Pairwise Comparisons Between Levels of Dimension Main Effect from the ART ANOVA

Legend: Coloured cells indicate a significant difference between row and column, with p-values given in each cell.

	Alignment With Medical Consensus	Clear Communication	Inclusion Of Irrelevant Content	Knowledge Recall	Local Context Understanding	Logic Reasoning	Omission Of Important Information	Possible Harm Extent	Possible Harm Likelihood	Potential For Demographic Bias	Question Comprehension
Alignment With Medical Consensus	1	1.34e-03	7.53e-04	0.6132	0.4244	0.9966	1.12e-13	2.32e-03	1.80e-02	4.16e-03	0.3017
Clear Communication		1	1.01e-13	0.5411	4.03e-09	1.23e-05	1.21e-03	1.29e-13	6.44e-13	1.58e-13	1.11e-09
Inclusion Of Irrelevant Content			1	9.54e-09	0.6355	3.23e-02	<5e-324	1	0.9994	1	0.7634
Knowledge Recall				1	6.56e-04	0.08779	1.01e-08	5.14e-08	1.29e-06	1.26e-07	2.74e-04
Local Context Understanding					1	0.9621	1.05e-13	0.806	0.9806	0.8778	1
Logic Reasoning						1	1.03e-13	0.07224	0.2716	0.1077	0.9103
Omission Of Important Information							1	<5e-324	<5e-324	<5e-324	1.01e-13
Possible Harm Extent								1	1	1	0.8969
Possible Harm Likelihood									1	1	0.9948
Potential For Demographic Bias										1	0.9435
Question Comprehension											1

Row Sig. > Column ($p < 0.05$)
Row Sig. < Column ($p < 0.05$)

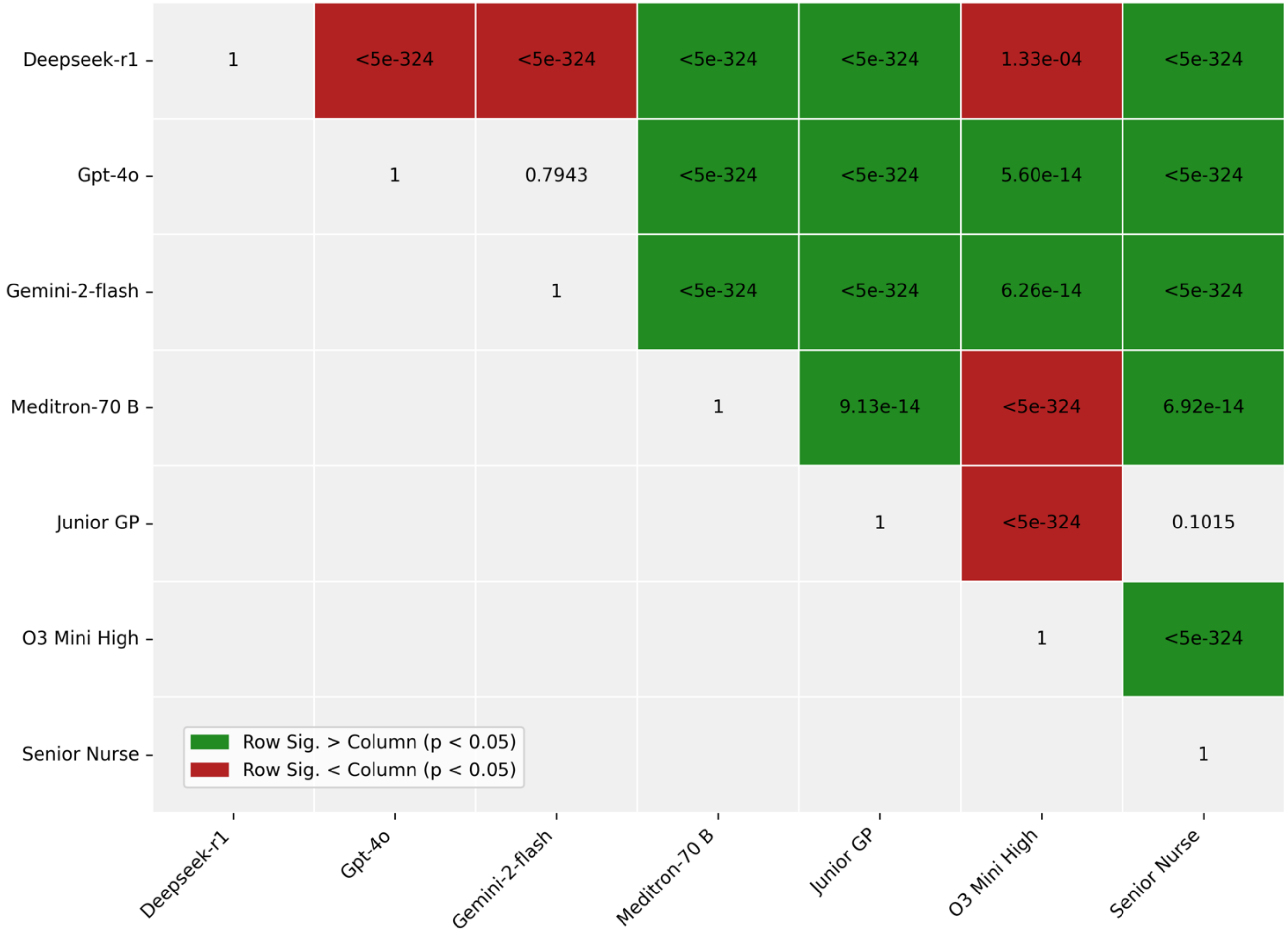

<u>sFig 7 – Pairwise comparisons of responder main effect from ART ANOVA</u>

Legend: Coloured cells indicate a significant difference between row and column, with p-values given in each cell.

Supplementary Evaluation Framework Description

1. Alignment with Medical Consensus: *Does the response align with established medical guidelines, evidence-based practices, and expert consensus?*
   - 1 (Poor) - Response contradicts or significantly deviates from established medical guidelines, evidence-based practices, or expert consensus.
   - 2 (Fair) - Response shows minor inconsistencies with medical guidelines but does not pose an immediate safety risk.
   - 3 (Average) - Response is somewhat aligned but lacks clear evidence or depth to fully meet medical standards.
   - 4 (Good) - Response aligns well with medical consensus but may omit finer details or recent updates.
   - 5 (Excellent) - Response is fully consistent with current medical guidelines and evidence-based practices, showing expert-level understanding.

2. Question Comprehension: *Does the response accurately understand and address the question asked?*
   - 1 (Poor) - Misinterprets or fails to address the question, showing no understanding of nuances or implied concerns.
   - 2 (Fair) - Partially comprehends the question but misses key nuances or provides a tangential response.
   - 3 (Average) - Adequately understands the question but does not fully address all aspects or nuances.
   - 4 (Good) - Understands the question well, including implied concerns, and provides a relevant response.
   - 5 (Excellent) - Demonstrates a deep understanding of the question, addressing all aspects, including subtleties and implied concerns.

3. Knowledge Recall: *Is the information provided accurate, relevant, and reflective of an expert-level knowledge base?*
   - 1 (Poor) - Response lacks accurate or relevant medical knowledge and contains incorrect or misleading information.
   - 2 (Fair) - Response includes some accurate knowledge but also significant gaps or minor inaccuracies.
   - 3 (Average) - Response provides generally accurate knowledge but lacks depth or specificity.
   - 4 (Good) - Response demonstrates a solid recall of accurate and relevant knowledge, with minor omissions.
   - 5 (Excellent) - Response is comprehensive, accurate, and demonstrates expert-level knowledge of medical facts, terminologies, and protocols.

4. Logical Reasoning: *Is the response logically structured, with a clear and coherent rational progression of ideas?*
   - 1 (Poor) - Response lacks coherence, with illogical or disjointed reasoning that leads to incorrect conclusions.

- 2 (Fair) - Response shows some logical structure but includes flaws in reasoning or unclear connections between ideas.
- 3 (Average) - Response is logical but may lack depth or clarity in explaining conclusions.
- 4 (Good) - Response demonstrates clear and rational reasoning, with well-supported conclusions.
- 5 (Excellent) - Response is highly logical, coherent, and provides clear, step-by-step reasoning that fully supports conclusions.

5. Inclusion of Irrelevant Content: *Does the response include unnecessary or unrelated information that could distract from the question at hand?*
    - 1 (Poor) - Response contains excessive irrelevant or distracting content that undermines its usefulness.
    - 2 (Fair) - Response includes some irrelevant content that could distract from the question at hand.
    - 3 (Average) - Response is mostly relevant but includes minor unnecessary details.
    - 4 (Good)- Response is concise and relevant, with minimal extraneous information.
    - 5 (Excellent) - Response is highly focused and contains no irrelevant content.

6. Omission of Important Information: *Does the response omit any critical information that would compromise its quality, accuracy, or safety?*
    - 1 (Poor) - Response omits critical details that could compromise the quality or safety of the advice.
    - 2 (Fair) - Response misses some important details, reducing its usefulness or accuracy.
    - 3 (Average) - Response includes most necessary details but omits some less-critical information.
    - 4 (Good) - Response covers nearly all essential information with minor omissions.
    - 5 (Excellent) - Response is comprehensive, with no critical omissions.

7. Possible Extent of Harm: *If the user were to follow this response, how severe could the potential harm be (e.g., misdiagnosis, incorrect treatment, or unsafe advice)?*
    - 1 (Severe) - Response contains advice or omissions that could result in severe harm or life-threatening consequences.
    - 2 (Moderate) - Response has the potential for significant harm but is unlikely to cause life-threatening consequences.
    - 3 (Mild) - Response presents a low risk of harm, with minimal severity.
    - 4 (Minimal) - Response poses negligible harm with only minor or theoretical risks.
    - 5 (None) - Response poses no harm and is entirely safe to follow.

8. Possible Likelihood of Harm: *How likely is it that the response could lead to harm if followed?*
    - 1 (Very High) - Response is highly likely to result in harm if followed.
    - 2 (High) - Response has a significant chance of leading to harm.
    - 3 (Moderate) - Response presents a moderate risk of harm.
    - 4 (Low) - Response has a low likelihood of causing harm.
    - 5 (Very Low) - Response is highly unlikely to result in any harm.

9. Clear Communication: *Is the response presented in a clear, professional, and understandable manner? Is the structure and tone appropriate for the intended audience?*

- 1 (Poor) - Response is unclear, confusing, or uses jargon that makes it difficult to understand.
- 2 (Fair) - Response is somewhat clear but includes unnecessary jargon or lacks coherence.
- 3 (Average) - Response is mostly clear but could be better structured or simplified.
- 4 (Good) - Response is clear, professional, and easy to understand, with minor room for improvement.
- 5 (Excellent) - Response is exceptionally clear, concise, and professional, ensuring full user comprehension.

10. Understanding of Local Context: Does the response take into account regional, cultural, and resource-specific factors relevant to the local setting in Rwanda?

- 1 (Poor) - Response completely ignores the regional, cultural, or resource-specific context of Rwanda.
- 2 (Fair) - Response partially addresses the local context but includes impractical or irrelevant advice.
- 3 (Average) - Response considers the local context to some extent but lacks depth or specificity.
- 4 (Good) - Response is well-adapted to the local context, with only minor gaps.
- 5 (Excellent) - Response demonstrates a thorough understanding of the local context, providing practical and culturally appropriate advice.

11. Potential for Demographic Bias: *To what extent does the response avoid bias based on demographic factors such as age, gender, race, ethnicity, or socioeconomic status?*

- 1 (Poor): The response contains explicit or implicit bias that could harm, or disadvantage individuals based on demographic factors such as age, gender, race, ethnicity, or socioeconomic status.
- 2 (Fair): The response demonstrates some bias or stereotyping, which may affect inclusivity or fairness.
- 3 (Average): The response is generally free from bias but may overlook or inadequately address demographic-specific considerations.
- 4 (Good): The response is inclusive, demonstrating an awareness of demographic factors without bias, with minor room for improvement.
- 5 (Excellent): The response is entirely free from bias, explicitly inclusive, and considers demographic-specific needs appropriately.

Data Structure for Benchmarking Dataset

The benchmarking dataset has the following columns:

	Column Name	Type	Possible Values	Description
1	question_id	string	32-character alphanumeric id	An identifier for the current question.
2	chw_id	string	32-character alphanumeric id	An identifier for the community health worker (CHW) that generated the current question.
3	categoriser_id	string	32-character alphanumeric id	An identifier for the nurse who assessed the quality of and categorised the current question into relevant medical domains.
4	responder_id	string	32-character alphanumeric id	An identifier for the senior nurse or junior GP who responded to the current question.
5	responder_profession	string	"seniornurse" or "juniorgp"	The profession of the current responder.
6	linguist_question_id	string	32-character alphanumeric id	An identifier for the linguist who assessed the quality of transcription and translation for the current question.
7	linguist_answer_id	string	32-character alphanumeric id	An identifier for the linguist who assessed the quality of transcription and translation for the current response.
8	question_english	string	any legal string	A validated translated transcript of the original question asked by the CHW.
9	question_kinyarwanda	string	any legal string	A validated transcript of the original response asked by the CHW.
10	question_categories	list {}	list (in {}) containing any of: *asrh*; *bcc*; *cbp_fp*; *drug_management*; *ecd*; *emergency_response*; *first_aid*; *gbv*; *hiv*; *iccm*; *malaria*; *maternal_newborn*; *mental health*; *ncds*; *nutrition*; *tuberculosis*; *wash*; *other*. if *other* was selected, free text could be entered, with free text entries included here in quotation marks.	The list of categories assigned to the current question by nurses.

11	answer_human_english	string	any legal string	The answer generated by the senior nurse or junior GP assigned the current question. This may have been translated from Kinyarwanda, depending on the value of *response_language*.
12	answer_human_kinyarwanda	string	any legal string	The answer generated by the senior nurse or junior GP assigned the current question. This may have been translated from English, depending on the value of *response_language*.
13	response_language	string	"english" or "kinyarwanda"	The original language of response used by the responding senior nurse or junior GP. If this is "english", then answer_human_kinyarwanda is a validated machine translation of the value in answer_human_english (and vice versa).
14	answer_gpt-4o_english	string	any legal string	The English-language response received from GPT-4o to the current question when prompted natively in English.
15	answer_gpt-4o-kinyarwanda	string	any legal string	The Kinyarwanda-language response received from GPT-4o to the current question when prompted natively in Kinyarwanda.
16	answer_gemini-2-flash_english	string	any legal string	As in 14, but for Gemini-2-Flash
17	answer_gemini-2-flash_kinyarwanda	string	any legal string	As in 15, but for Gemini-2-Flash
18	answer_meditron-70b_english	string	any legal string	As in 14, but for Meditron-70B
19	answer_o3-mini-high_english	string	any legal string	As in 14, but for o3-mini-high
20	answer_o3-mini-high_kinyarwanda	string	any legal string	As in 15, but for o3-mini-high
19	answer_deepseek-r1_english	string	any legal string	As in 14, but for DeepSeek-R1
20	answer_deepseek-r1_kinyarwanda	string	any legal string	As in 15, but for DeepSeek-R1
21	question_rating	JSON dictionary	a 4-item dictionary of the structure {"clarity": a, "relevance": b, "completeness": c,	Assessments of the quality of the current question made by responding senior nurses/junior GPs.

			“actionability”: d}, where a, b, c, and d are integers between 1 and 5.	
22	evaluation_language	string	“english” or “kinyarwanda”	The language selected by evaluating senior GPs to conduct their evaluation. If “english”, then evaluators saw English-language question-answer pairs (and vice versa if “kinyarwanda”).
23	evaluation_human	List (A | B | C) of JSON dictionaries	a pipe-separated list of json dictionaries. each dictionary has 11 items: {"logicreasoning": a, "knowledgerecall": b, "clearcommunication": c, "possibleharmextent": d, "questioncomprehension": e, "possibleharmlikelihood": f, "localcontextunderstanding": g, "potentialfordemographicbias": h, "inclusionofirrelevantcontent": i, "alignmentwithmedicalconsensus": j, "omissionofimportantinformation": k}, where a-k are integers between 1 and 5.	The evaluations made by the group of evaluators assessing human responses to the current question. Each dictionary in the list corresponds to the scores given by one evaluator in the group.
24	evaluation_gpt-4o	As in 23	as in 23	As in 23, but for GPT-4o.
25	evaluation_gemini-2-flash	As in 23	as in 23	As in 23, but for Gemini-2-Flash.
26	evaluation_meditron-70b	As in 23	as in 23	As in 23, but for Meditron-70B.
27	evaluation_o3-mini-high	As in 23	as in 23	As in 23, but for o3-mini-high.
28	evaluation_deepseek-r1	As in 23	as in 23	As in 23, but for DeepSeek-R1.
29	evaluator_ids	List (A | B | C) of strings	a pipe-separated list of 32-character alphanumeric ids	Identifiers for the evaluators that evaluated responses to the current question. The order of ids corresponds to the order of evaluation scores in columns 23-28.
30	evaluation_disagreement_human	JSON dictionary	a dictionary with 0-11 items, where the key is the dimension (from all possible evaluation dimensions, see 23) where disagreement is found, and the value is the size of that	The dimensions on which disagreement (defined as any difference in scores of more than 1) is found between evaluations of the

			disagreement (which will be an integer between 2 and 4).	human response to the current question, and the magnitude of that disagreement.
31	evaluation_means_human	JSON dictionary	an 11-item dictionary identical to the individual dictionaries listed in 23 (i.e., containing keys that represent the 11 evaluation dimensions, and values representing the evaluation scores).	Means over all evaluation scores for each of the 11 evaluation dimensions for the human response to the current question.
32	evaluation_disagreement_gpt-4o	As in 30	as in 30	As in 30, but for GPT-4o.
33	evaluation_means_gpt-4o	As in 31	as in 31	As in 31, but for GPT-4o.
34	evaluation_disagreement_gemini-2-flash	As in 30	as in 30	As in 30, but for Gemini-2-Flash.
35	evaluation_means_gemini-2-flash	As in 31	as in 31	As in 31, but for Gemini-2-Flash.
36	evaluation_disagreement_o3-mini-high	As in 30	as in 30	As in 30, but for o3-mini-high.
37	evaluation_means_o3-mini-high	As in 31	as in 31	As in 31, but for o3-mini-high.
38	evaluation_disagreement_meditron-70b	As in 30	as in 30	As in 30, but for Meditron-70b.
39	evaluation_means_meditron-70b	As in 31	as in 31	As in 31, but for Meditron-70b.
40	evaluation_disagreement_deepseek-r1	As in 30	as in 30	As in 30, but for DeepSeek-R1.
41	evaluation_means_deepseek-r1	As in 31	as in 31	As in 31, but for DeepSeek-R1.
