# Supplementary material for: Large language models for frontline healthcare support in low-resource settings
Source: Nat Health. 2026 Feb 6;1(2):191–7. doi: 10.1038/s44360-025-00038-1 (PMC12880909; doi:10.1038/s44360-025-00038-1)
Supplement: Supplementary file 2 — Reporting Summary [file 44360_2025_38_MOESM2_ESM.pdf]

nature portfolio

Corresponding author(s): Bilal A. Mateen

Last updated by author(s): Nov 10, 2025

Reporting Summary

Nature Portfolio wishes to improve the reproducibility of the work that we publish. This form provides structure for consistency and transparency in reporting. For further information on Nature Portfolio policies, see our Editorial Policies and the Editorial Policy Checklist.

Statistics

For all statistical analyses, confirm that the following items are present in the figure legend, table legend, main text, or Methods section.

n/a	Confirmed	
☐	☒	The exact sample size (*n*) for each experimental group/condition, given as a discrete number and unit of measurement
☐	☒	A statement on whether measurements were taken from distinct samples or whether the same sample was measured repeatedly
☐	☒	The statistical test(s) used AND whether they are one- or two-sided<br>*Only common tests should be described solely by name; describe more complex techniques in the Methods section.*
☐	☒	A description of all covariates tested
☐	☒	A description of any assumptions or corrections, such as tests of normality and adjustment for multiple comparisons
☐	☒	A full description of the statistical parameters including central tendency (e.g. means) or other basic estimates (e.g. regression coefficient) AND variation (e.g. standard deviation) or associated estimates of uncertainty (e.g. confidence intervals)
☐	☒	For null hypothesis testing, the test statistic (e.g. *F*, *t*, *r*) with confidence intervals, effect sizes, degrees of freedom and *P* value noted<br>*Give P values as exact values whenever suitable.*
☒	☐	For Bayesian analysis, information on the choice of priors and Markov chain Monte Carlo settings
☒	☐	For hierarchical and complex designs, identification of the appropriate level for tests and full reporting of outcomes
☒	☐	Estimates of effect sizes (e.g. Cohen's *d*, Pearson's *r*), indicating how they were calculated

Our web collection on statistics for biologists contains articles on many of the points above.

Software and code

Policy information about availability of computer code

Data collection	Data collection relied on a closed-source digital platform (Mbaza) developed by Digital Umuganda specifically for this project. Source code for the platforms developed will be made available by Digital Umuganda upon request.
Data analysis	A set of python and R scripts were written to analyse the data collected. All analysis code is available here (and all packages used are detailed in the requirements.txt file in the link provided): https://github.com/PATH-AI-Initiative/RwandaBenchmarking<br><br>The following python and R packages/libraries were used for data processing and analysis:<br>Python (v3.13.3): pandas (v2.2.3); matplotlib (v3.10.3); numpy (v2.2.6); seaborn (v0.13.2); scipy (v1.15.3); tiktoken (v0.9.0); transformers (v4.52.4); statsmodels (v0.14.4)<br>R (v4.5.0): ARTool (v0.11.2); dplyr (v1.1.4); emmeans (v1.10.7); readr (v2.1.5); stringr (v1.5.1); ggplot2 (v3.5.2)

For manuscripts utilizing custom algorithms or software that are central to the research but not yet described in published literature, software must be made available to editors and reviewers. We strongly encourage code deposition in a community repository (e.g. GitHub). See the Nature Portfolio guidelines for submitting code & software for further information.

Data

Policy information about availability of data

All manuscripts must include a data availability statement. This statement should provide the following information, where applicable:

- Accession codes, unique identifiers, or web links for publicly available datasets
- A description of any restrictions on data availability
- For clinical datasets or third party data, please ensure that the statement adheres to our policy

The subset of 524 questions, answers, and individual evaluation results that comprise this benchmarking study is available via Figshare (https://doi.org/10.6084/m9.figshare.29213147). The data structure for the whole dataset is provided as supplementary material 2. The full dataset has been donated to the Rwanda Biomedical Centre (RBC), the parastatal delivery arm of the Rwandan Ministry of Health, and is hosted in a secure data environment. It will be made available to researchers on request and based on an assessment of 'fair value exchange' by stakeholders, to ensure that the indigenous population that generated the information benefits from its exploitation. This arrangement was specifically designed to ensure adherence to the CARE principles. The Centre for the Fourth Industrial Revolution, as the innovation lab for the Rwandan Government, serves as the primary point of contact for researchers seeking to access this data. Prospective users should contact 'info@c4ir.rw' to request access. Requests for access will be responded to within 1 month

Research involving human participants, their data, or biological material

Policy information about studies with human participants or human data. See also policy information about sex, gender (identity/presentation), and sexual orientation and race, ethnicity and racism.

Reporting on sex and gender	Neither sex nor gender were recorded nor analysed in this research, with the exception of recording the (self-reported) gender of community health workers, nurses, and clinicians who participated in data generation. That said, our evaluation of model output did prompt evaluators to consider whether responses avoided "bias based on demographic factors such as age, gender, race, ethnicity, or socioeconomic status". Performance for all responding clinicians and models on this metric are reported in the manuscript. Population characteristics for the participating CHWs, nurses, and doctors can be found in the Supplementary Materials (sTables 1-4).
Reporting on race, ethnicity, or other socially relevant groupings	Neither race, ethnicity, nor any other socially relevant groupings were recorded nor analysed through this research. Again, however, our evaluation of model output did prompt evaluators to consider whether responses avoided "bias based on demographic factors such as age, gender, race, ethnicity, or socioeconomic status". Performance for all responding clinicians and models on this metric are reported in the manuscript.
Population characteristics	The only population characteristics used as a covariate in our analysis were:<br>1) the profession of the clinicians generating responses to community health worker questions. This was either "junior GP" (general practitioners with 2 to 5 years of clinical experience) or "senior nurse" (nurses with at least five years of clinical experience)<br><br>2) the language spoken by those clinicians when generating responses – this was either English or Kinyarwanda.<br><br>These population characteristics were chosen given that one's clinical profession is likely to affect one's ability to generate accurate and robust responses to questions posed by community health workers, and given that we expected model performance to vary by language, and so we planned an equivalent analysis of human responses.
Recruitment	Community health workers, nurses, general practitioners, and specialist clinicians were recruited by leveraging Digital Umuganda and the Centre for the Fourth Industrial Revolution's existing networks within the Rwandan healthcare system. CHWs were selected and recruited based on recommendations made by the Rwanda Biomedical Centre (who implement and manage the community health programme), and all clinicians recruited were done so by senior clinicians (based primarily at the Butaro District Hospital) with whom Digital Umuganda had worked previously. For further detail, see the Method section of the main manuscript.<br><br>Our recruitment method is naturally biased in favour of the networks of clinicians known to us, and there is likely some degree of self-selection bias, in that more ardent clinicians are more likely to take on additional work. However, in our view this kind of bias only makes our research more conservative: more enthusiastic clinicians may provide more complete answers to queries sent to them by community health workers, providing a better representation of best-in-class human responses. Despite this, several of our models outperformed human clinicians across the board. In addition, the magnitude of the difference here is unlikely to be accounted for by biases in recruitment, which we would expect to have much smaller effects.
Ethics oversight	The study was deemed exempt from review by the Rwanda National Ethics Committee. PATH's research determination committee also reviewed the scope and confirmed it was not human subjects research subject to IRB approval.

Note that full information on the approval of the study protocol must also be provided in the manuscript.

Field-specific reporting

Please select the one below that is the best fit for your research. If you are not sure, read the appropriate sections before making your selection.

☒ Life sciences ☐ Behavioural & social sciences ☐ Ecological, evolutionary & environmental sciences

Life sciences study design

All studies must disclose on these points even when the disclosure is negative.

Sample size	No sample size calculation was performed, since the primary aim of this research was to generate a useful dataset, not to sufficiently power any particular analysis. We set reasonable targets for vignette generation by participating nurses, and used the funds available to us to evaluate as many responses via human evaluation as was feasible. All subsequent sample sizes (i.e., the number of question/answer pairs included in the final dataset) flowed from that point.
Data exclusions	CHW-generated vignettes were screened for their quality by local nurses, who would exclude vignettes from the final dataset if: they failed to follow the SBAR tool used during vignette generation (see Methods for more detail); they lacked sufficient informati\ason for a clinician to provide a sound response to the question posed; or if the audio recording was incomplete or inaudible. A total of 1534 vignettes were rejected on these bases (of a total 7143 vignettes assessed).<br><br>Subsequently, clinicians were instructed to generate responses to the first 5000 of the 5609 questions that passed quality assurance. There was some delay in stopping response generation once the 5000-question target had been met, resulting in a final sample of 5422. The 187 remaining questions were excluded from the final, complete dataset for lack of a clinician-generated response.<br><br>Finally, 18 question-answer pairs from the final dataset were excluded due to unresolved disagreement between human evaluators (see Replication section below for more detail).
Replication	To verify the reproducibility of human assessment of model and clinician responses to CHW-generated queries, we had two independent evaluators review every response to every question. Evaluators assessed every response on 11 dimensions, providing a score on a 5-point Likert scale for each dimension. Any difference greater than 1 between scores on any dimension for a given response to a given question was classed as disagreement. Independent evaluators then convened to resolve any disagreements and select an appropriate score. However, disagreement remained unresolved on 18 question-answer pairs, which were excluded from all analyses of the human evaluation.
Randomization	There was no allocation to conditions in this study, since the primary factors of interest were not ones in need of allocation. The exact question-answer pairs evaluated by our two teams of human evaluators, however, were randomly selected and distributed.
Blinding	Evaluators were blinded to the source of the responses they evaluated. That is, they did not know whether a given response came from a human clinician, from GPT-4o, from Gemini-2-Flash etc. Evaluators assessed all 6 responses to a given question in a random order with no information given on the source of the responses presented.

Reporting for specific materials, systems and methods

We require information from authors about some types of materials, experimental systems and methods used in many studies. Here, indicate whether each material, system or method listed is relevant to your study. If you are not sure if a list item applies to your research, read the appropriate section before selecting a response.

Materials & experimental systems

n/a	Involved in the study	
☒	☐	Antibodies
☒	☐	Eukaryotic cell lines
☒	☐	Palaeontology and archaeology
☒	☐	Animals and other organisms
☒	☐	Clinical data
☒	☐	Dual use research of concern
☒	☐	Plants

Methods

n/a	Involved in the study	
☒	☐	ChIP-seq
☒	☐	Flow cytometry
☒	☐	MRI-based neuroimaging

Plants

Seed stocks	*Report on the source of all seed stocks or other plant material used. If applicable, state the seed stock centre and catalogue number. If plant specimens were collected from the field, describe the collection location, date and sampling procedures.*
Novel plant genotypes	*Describe the methods by which all novel plant genotypes were produced. This includes those generated by transgenic approaches, gene editing, chemical/radiation-based mutagenesis and hybridization. For transgenic lines, describe the transformation method, the number of independent lines analyzed and the generation upon which experiments were performed. For gene-edited lines, describe the editor used, the endogenous sequence targeted for editing, the targeting guide RNA sequence (if applicable) and how the editor was applied.*
Authentication	*Describe any authentication procedures for each seed stock used or novel genotype generated. Describe any experiments used to assess the effect of a mutation and, where applicable, how potential secondary effects (e.g. second site T-DNA insertions, mosiacism, off-target gene editing) were examined.*
